# Enhanced Gravitational Search Optimization with Hybrid Deep Learning Model for COVID-19 Diagnosis on Epidemiology Data

**DOI:** 10.3390/healthcare10071339

**Published:** 2022-07-19

**Authors:** Mahmoud Ragab, Hani Choudhry, Amer H. Asseri, Sami Saeed Binyamin, Mohammed W. Al-Rabia

**Affiliations:** 1 Information Technology Department, Faculty of Computing and Information Technology, King Abdulaziz University, Jeddah 21589, Saudi Arabia; 2 Centre for Artificial Intelligence in Precision Medicines, King Abdulaziz University, Jeddah 21589, Saudi Arabia; hchoudhry@kau.edu.sa (H.C.); ahasseri@kau.edu.sa (A.H.A.); 3 Mathematics Department, Faculty of Science, Al-Azhar University, Nasr City, Cairo 11884, Egypt; 4 Biochemistry Department, Faculty of Science, King Abdulaziz University, Jeddah 21589, Saudi Arabia; 5 Computer and Information Technology Department, The Applied College, King Abdulaziz University, Jeddah 21589, Saudi Arabia; ssbinyamin@kau.edu.sa; 6 Department of Medical Microbiology and Parasitolog, Faculty of Medicine, King Abdulaziz University, Jeddah 21589, Saudi Arabia; mwalrabia@kau.edu.sa; 7 Health Promotion Center, King Abdulaziz University, Jeddah 21589, Saudi Arabia

**Keywords:** COVID-19, disease detection, health promotion, fusion model, hybrid deep learning, epidemiology data, parameter optimization

## Abstract

Effective screening provides efficient and quick diagnoses of COVID-19 and could alleviate related problems in the health care system. A prediction model that combines multiple features to assess contamination risks was established in the hope of supporting healthcare workers worldwide in triaging patients, particularly in situations with limited health care resources. Furthermore, a lack of diagnosis kits and asymptomatic cases can lead to missed or delayed diagnoses, exposing visitors, medical staff, and patients to 2019-nCoV contamination. Non-clinical techniques including data mining, expert systems, machine learning, and other artificial intelligence technologies have a crucial role to play in containment and diagnosis in the COVID-19 outbreak. This study developed Enhanced Gravitational Search Optimization with a Hybrid Deep Learning Model (EGSO-HDLM) for COVID-19 diagnoses using epidemiology data. The major aim of designing the EGSO-HDLM model was the identification and classification of COVID-19 using epidemiology data. In order to examine the epidemiology data, the EGSO-HDLM model employed a hybrid convolutional neural network with a gated recurrent unit based fusion (HCNN-GRUF) model. In addition, the hyperparameter optimization of the HCNN-GRUF model was improved by the use of the EGSO algorithm, which was derived by including the concepts of cat map and the traditional GSO algorithm. The design of the EGSO algorithm helps in reducing the ergodic problem, avoiding premature convergence, and enhancing algorithm efficiency. To demonstrate the better performance of the EGSO-HDLM model, experimental validation on a benchmark dataset was performed. The simulation results ensured the enhanced performance of the EGSO-HDLM model over recent approaches.

## 1. Introduction

COVID-19 or Coronavirus is a recent disease caused by severe acute respiratory syndrome coronavirus 2 (SARS-CoV-2) [1]. In February 2021, over 106 billion SARS-CoV-2 infections and a mortality rate of above 2.3 billion were recorded globally, in the worst epidemic to have troubled humans since the Spanish flu occurred in 1918, and has consequently overwhelmed global healthcare systems resulting in serious economic disturbance [2]. In order to combat this infectious disease, doctors and researchers have tirelessly attempted to gain further knowledge and develop technical devices for the diagnosis of this disease. Attempts include the advancement of drugs and vaccines [3], the formation of epidemiologic methods for forecasting the spread of the disease among people [4], the advancement of mobile device apps to track patients who are infected, the enhancement of policies, and the implementation of novel technologies for managing the capacities and resources in clinics [5]. Owing to the increasing prevalence of coronavirus infection worldwide, many studies analyzed various prospects of this infection. Research includes recognizing the methods of virus detection, the origin of coronavirus along with examining its gene series, early cases in the countries infected, forecasting COVID-19 cases in the epidemiologic pandemic, and patient information [6].

Machine learning (ML) procedures first accumulate data individually, i.e., from various sources [7], followed by fixing the preprocessed data to solve data-oriented problems and minimize space size by erasing incorrect data and selecting relevant data. However, in some cases, the dataset value may differ for the system in making decisions, thus, ML methods were devised with the help of other ideas such as probability statistics and theory control for examining data and deriving useful and original information, concealed information, or patterns from past knowledge [8]. Lastly, the performance assessments of the methods is conducted and model optimization concludes the process, which enhances the method with the help of novel rules and a dataset. ML approaches are utilized in various sectors such as traffic management, medicine, education, manufacturing and production, robotics, engineering, and so on [9,10,11]. Currently, ML methods are utilized in the examination of high dimensional bio-medical structured and unstructured datasets. Malaria Diagnosis, diabetes risk assessment, typhoid, and vascular diseases classification, and genomic and genetic data studies are a few instances of the bio-medical use of ML approaches.

The authors in [12] developed a novel image processing-based approach for the health care system called “C19D-Net”, for the detection of “COVID-19” in CXR images to assist radiologist in enhancing COVID-19 classification performance. It uses the InceptionV4 model with a multi-SVM classifier for COVID-19 diagnosis in distinct class labels. In [13], the researchers present a solution to analyze the COVID-19 pandemic dataset. Specifically, the solution focuses on analyzing useful knowledge and valuable information (for example, frequency, distribution, and pattern) of healthcare statuses and characteristics in populations. The availability of knowledge and information assists users (for example, civilians, and researchers) in better understanding the disease, and playing an active role in combating, fighting, or controlling the disease. Li et al. [14] performed a qualitative and quantitative assessment of Chinese social networking media posts originating in Wuhan City on the Chinese microblogging platform Weibo during the earlier stage of the COVID-19 epidemic.

The researchers in [15] reviewed the available approaches while anticipating the challenges and difficulties in the improvement of a data-driven strategy to combat the COVID-19 outbreak. A 3M-analysis was presented, which included making, monitoring, and exhibiting decisions. The emphasis was placed on the potential of a familiar data-driven scheme to tackle various problems which increased following the outbreak; firstly, by forecasting and monitoring the spread of the epidemic; secondly, by assessing the efficiency of government decisions; and lastly, by making appropriate decisions. Every step of the roadmap may be exhaustive for an analysis of consolidated theoretic outcomes and the potential applications in the COVID-19 context. Supervised ML methods for COVID-19 infection were designed in [16] with learning algorithms including DT, LR, NB, ANN, and SVM using epidemiology labeled data for negative and positive COVID-19 cases. A correlation coefficient (CC) analysis between independent features and dependent features was performed to determine stronger relationships among every dependent and independent feature of the prior dataset to develop the model.

The researchers in [17] processed available information on US states to create an incorporated dataset on potential factors which cause the epidemic to spread. Then, the researchers made use of the supervised ML approach to reach a consensus and rank the crucial factor. The author performed a regression analysis to identify the crucial pre-lockdown factors that affected post-lockdown contamination and death to inform lockdown-related policy making. Kim et al. [18] aimed to examine the relationship between obesity, as determined by the body mass index (BMI), with mortality and morbidity owing to COVID-19. Information from 5628 approved COVID-19 patients were gathered by the Center for Disease Control and Prevention of Korea. The odds ratio (OR) of diabetes and morbidity in the BMI groups was examined using LR models attuned for similar covariates. Hawkins et al. [19] designed a nationwide COVID-19 dataset at the county level that was coupled with the Distressed Communities Index (DCI) and their constituent metrics of socio-economic status.

This study developed an Enhanced Gravitational Search Optimization with Hybrid Deep Learning Model (EGSO-HDLM) for COVID-19 diagnosis using epidemiology data. The proposed EGSO-HDLM model employs a hybrid convolutional neural network with gated recurrent unit based fusion (HCNN-GRUF) model. In addition, the hyperparameter optimization of the HCNN-GRUF model was improved by the use of the EGSO algorithm, which was derived by including the concepts of cat map and the traditional GSO algorithm. To assure the enhanced performance of the EGSO-HDLM model, an extensive set of experimental validation processes on a benchmark dataset was performed. In short, the paper’s contribution is summarized as follows.

- An intelligent EGSO-HDLM model which includes pre-processing, HCNN-GRUF classification, and EGSO-based parameter optimization is presented. To the best of our knowledge, the EGSO-HDLM model has not previously been presented in the literature.
- Hyperparameter optimization of the HCNN-GRU model with the EGSO algorithm using cross-validation helps to boost the predictive outcome of the EGSO-HDLM model for unseen data.
- Validating the performance of the proposed EGSO-HDLM model on a benchmark dataset from the Kaggle repository.

## 2. The Proposed Model

**Figure 1:**
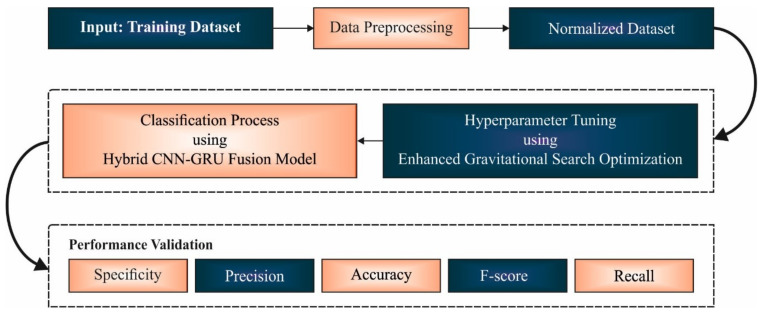
Overall process of EGSO-HDLM technique.

In this study, a new EGSO-HDLM model was designed for the identification and classification of COVID-19 using epidemiology data. Primarily, the presented EGSO-HDLM model applied data normalization to pre-process the actual data. Then, the HCNN-GRUF model was applied for the classification process. Moreover, the hyperparameter optimization of the HCNN-GRUF model was improved by the use of the EGSO algorithm. Figure 1 depicts the overall process of the EGSO-HDLM algorithm.

### 2.1. Data Pre-processing

When the numerical processing was executed, a huge variance in magnitude amongst the values became apparent. This condition can cause problems such as minimal convergence of networks and saturation of neuron outcomes [20]; therefore, normalized original data were required. The following Min-Max normalized approach (Equation (1)) can be used for normalizing the data to intervals of zero and one:

(1) $$x^{*}=\frac{x-x_{\;\min\;}}{x_{\;\max\;}-x_{\;\min\;}}.\;$$

where $x^{*}$ signifies the normalized data, x represents the original data, $x_{\min}$ implies the minimal data values from the present element, and $x_{\max}$ denotes the maximal data value from the present elements.

### 2.2. Design of HCNN-GRUF Based Classification

CNN [21] is a hierarchical FFNN approach that is dependent upon convolutional as well as pooling functions. Convolution was utilized to extract local features for further processing with subsequent layers. Primarily, the resultant input layers are convoluted with several n×h filters (convolutional kernel). Next, the resultant convolutional layer was created by linking the outcomes of the convolutional function that differs with filter size h.

(2) $$m_{i}=f\left(w\cdot x_{i+h-1}+b\right)$$

(3) $$M=\left[m_{1},\;m_{2},\;m_{3},\;\ldots ,\;m_{l-h+1}\right]$$

**Figure 2:**
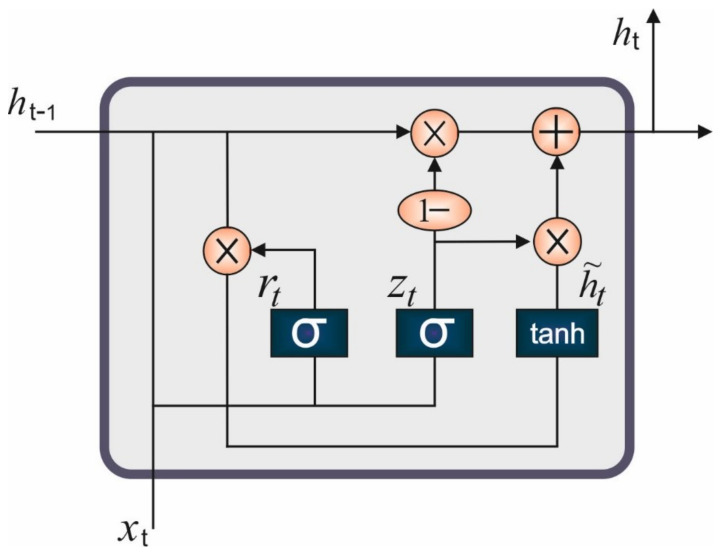
Structure of GRU Model.

In Equation (2), $m_{i}$ refers the ith feature extracted with convolutional function, f denotes the non-linear function, w signifies the weighting of filters, h denotes the filtering window size, and b implies the bias. A combination of GRU and LSTM was applied for a gated recurrent neural network (RNN) which remembers a long order of data and thus decreases data loss. Related to LSTM, GRU decreases the amount of gated units that reduces the processing time but preserves the accuracy. GRU has two gated sub-units, namely reset as well as update gates. In every moment, the GRU receives the existing as well as hidden states in the preceding moment with their update gate, and defines the activation state of their individual neurons. Additionally, the reset gate receives both states and defines if any input data are forgotten. Figure 2 depicts the framework of the GRU technique. Input at the present moment is then integrated with the weighted and resultant reset gate to obtain the memory contents at the present moment with activation functions. Next, the upgrade gate obtains the memory content at the present moment and hidden state in the preceding moment for determining the output and hidden state at the present moment. The function of GRU is summarized in the following formulas.

(4) $$z_{t}=\sigma \left(W^{\left(z\right)}\cdot x_{t}+U^{\left(z\right)}\cdot h_{t-1}\right)$$

(5) $$r_{t}=\sigma \left(W^{\left(z\right)}\cdot x_{t}+U^{\left(r\right)}\cdot h_{t-1}\right)$$

(6) $$h_{t}^{\prime }=tanh\left(W\cdot x_{t}+r_{t}\cdot U\cdot h_{t-1}\right)$$

(7) $$h_{t}=z_{t}\cdot h_{t-1}+\left(1-z_{t}\right)\cdot h_{t}^{\prime }$$

In Equation (4), $z_{t}$ signifies the upgrade gate, $W^{\left(z\right)}$ and $U^{\left(z\right)}$ imply the weighting of $z_{t},$ σ stands for the activation function, $x_{t}$ indicates the input at present moment, and $h_{t-1}$ represents the hidden output in the preceding moment. $r_{t}$ stands for the reset gate, and $W^{\left(r\right)}$ and $U^{\left(r\right)}$ refer the weighting of $r_{t}.$ $h_{t}^{\prime }$ defines the memory satisfied at the present moment, tanh implies the activation functions, and W and U represent the weighting at the present moment. $h_{t}$ defines the outcome at the present moment. Pooling is the procedure of extracting data for the purpose of reducing the size of the resultant GRU layer. The maximal pooling approach was utilized for extracting the maximal vector value of all the inputs as the result of pooling layers.

(8) $$\hat{m}=\;\max\;\left\{M\right\}$$

(9) $$z=\left[{\hat{m}}_{1},\;{\hat{m}}_{2},\;{\hat{m}}_{3},\;\ldots \ldots ,\;{\hat{m}}_{k}\right]$$

where M denotes the typical vector in the GRU to pooling layers, $\hat{m}$. implies the maximal of M, z signifies the output resultant in the pooling layer, and k denotes the amount of features input to pooling layers. The fusion layer was planned to merge more than 2 layers of tensors; during this case, it integrates several tensors in the polling layer as opposed to one tensor. It can be executed by the “Concatenate” approach that receives the final bit as axis, and splices all the resultant polling layers for creating the result of this layer.

(10) $$b=\left[a_{1},\;a_{2}\right]$$

In Equation (10), b refers the output of this layer, aa denotes the resultant in the polling layers. The softmax classification is usually employed for several classifier problems. The fully connected (FC) layer was utilized to connect an input in the fusion layer and the value of all the neurons was computed utilizing the softmax function. Additionally, the maximal value of every neuron output was obtained as the classifier outcome.

(11) $$\hat{p}=soft\max\;\left(W\cdot x+b\right)$$

(12) $$y=argmax\left({\hat{p}}_{y}\right)$$

In Equation (11), ${\hat{p}}_{y}$ signifies the probability of y, forecast labels. W and b denote the weights as well as biases. In Equation (12), y denotes the last forecast label.

### 2.3. Hyperparameter Tuning

At this stage, the hyperparameter tuning of the HCNN-GRUF model can be tuned by the design of the EGSO algorithm. Rashedi [22] developed a GSO algorithm, which is an optimization algorithm that gained popularity in the past few decades. It depends on the second law of motion, as illustrated in Equation (15), and the law of gravity, as demonstrated in Equation (13). Additionally, it depends on the physical concepts of passive gravitational mass, inertial mass, and active gravitational mass. The second law of motion states that once a force (F) is applied to all the particles, the (a) acceleration is defined by the mass (M) and force. The law of gravity says that all the particles attract all the other particles with a gravitational force (F). The gravitational force (F) between these particles is directly proportionate to the product of ($M_{1}$ and $M_{2}$) masses and inversely proportionate to the square of $\left(R^{2}\right)$ distance.

(13) $$F=G\frac{M_{1}M_{2}}{R^{2}}$$

G indicates the gravitational constant that reduces with increasing time, and it is evaluated by the following expression:

(14) $$G\left(t\right)=G\left(t_{0}\right)\times {\left(\frac{t_{0}}{t}\right)}^{\beta }\;,\;\beta <1$$

(15) $$a=\frac{F}{M}$$

The GSA algorithm is briefly described in the following steps:

- Step one: Initialization

An isolated scheme with N number of particles is assumed, where the location of i-th particles is represented as follows:

(16) $$P_{i}=\left(p_{i}^{1},\;p_{i}^{2},\;\ldots ,\;p_{i}^{d},\;\ldots ,\;p_{i}^{N}\right)\;for\;i=1,2,3,\ldots ,N$$

In Equation (16), $p_{i}^{d}$ indicates the location of i-th particles in dimension d.

- Step two: Fitness assessment of particle

Here, the best and worst fitness values are evaluated using Equations (17) and (18) correspondingly. Minimization and maximization problems are evaluated by Equations (19) and (20).

(17) $$worst\left(t\right)=\underset{j,\left\{1,\ldots ,N\right\}}{\max}fitness_{j}\left(t\right)$$

(18) $$best\;\left(t\right)=\underset{j,\left\{1,\ldots ,N\right\}}{\min}fitness_{j}\left(t\right)$$

(19) $$worst\left(t\right)=\underset{j,\left\{1,\ldots ,N\right\}}{\min}fitness_{j}\left(t\right)\;$$

(20) $$best\;\left(t\right)=\underset{j,\left\{1,\ldots ,N\right\}}{\max}fitness_{j}\left(t\right)$$

In the equation, $fitness_{j}\left(t\right)$ refers to the fitness of j-th particles at t time.

- Step three: Evaluate the $G\left(t\right)$ gravitational constant

Here, the $G\left(t\right)$ gravitational constant at t time can be evaluated using Equation (21):

(21) $$G\left(t\right)=G_{0}\times \left(1-\frac{t}{t_{\;\max\;}}\right)$$

where $G_{0}$ is the primary value of the gravitational constant initialized at a random fashion, t indicates the present time, and $t_{\max\;}$ denotes the overall time.

- Step four: Upgrade the inertial and gravitational mass

Here, the gravitational and inertia mass are upgraded with the help of the fitness function. Equality of gravitational and inertia masses is assumed, and the masses value is evaluated using the following expression:

(22) $$M_{ii}=M_{pi}=M_{ai}=M_{i}\;for\;i=1,2,3,\ldots N$$

(23) $$m_{i}\left(t\right)=\frac{fitness_{i}\left(t\right)-worst\left(t\right)}{best\left(t\right)-worst\left(t\right)}$$

(24) $$M_{i}\left(t\right)=\frac{m_{i}\left(t\right)}{\sum_{j=1}^{N}m_{j}\left(t\right)}$$

In the expression, $fitness_{i}\left(t\right)$ represent the fitness of i-th particles at time and t, $M_{i}\left(t\right)$ denotes the mass of i-th particles at t time.

- Step five: Calculate the overall force

Here, the complete force $F_{i}^{d}\left(t\right)$ that is exerted on the i-th particles in a d-th dimension at t time is evaluated by the following equation:

(25) $$F_{i}^{d}\left(t\right)=\overset{\mathrm{kb}est}{\underset{j=1,j\neq i}{\sum }}rand_{j}F_{ij}^{d}\left(t\right)$$

In Equation (25), $rand_{j}$ represent a random integer ∈ $\left[0,\;1\right]$, and kbest is the set of initial particles with the biggest masses and the best fitness value. The force applied from mass ‘*j*’ on mass ‘*i*’ at ‘*t*’ time can be expressed as $F_{ij}^{d}\left(t\right)$ and is evaluated using Equation (26):

(26) $$F_{ij}^{d}\left(t\right)=G\left(t\right)\frac{M_{pi}\left(t\right)\times M_{aj}\left(t\right)}{R_{ij}\left(t\right)+\tau }\left(p_{j}^{d}\left(t\right)-p_{i}^{d}\left(t\right)\right)$$

Consider that $M_{pi}$ refers to the passive gravitational mass correlated to i-th particles, $Ma_{j}$ denotes the active gravitational mass interrelated to j-th particles. τ indicates a smaller positive constant to avoid division by 0, and $R\left(t\right)$ indicates the Euclidian distance between i-th and j-th particles:

(27) $$R_{ij}\left(t\right)=\|p_{i}\left(t\right),p_{j}\left(t\right){\|}_{2}$$

- Step six: Calculate the acceleration and velocity

Here, $F_{i}^{d}\left(t\right)$, the acceleration of i-th particles; $a_{i}^{d}\left(t\right)$ at t time in the d direction; and the velocity of i-th particles in the d-th direction, $u_{i}^{d}\left(t+1\right)$, are evaluated by:

(28) $$a_{i}^{d}\left(t\right)=\frac{F_{i}^{d}\left(t\right)}{M_{ii}\left(t\right)}$$

(29) $$u_{i}^{d}\left(t+1\right)=rand_{i}\times u_{i}^{d}\left(t\right)+a_{i}^{d}\left(t\right)$$

From the expression, $M_{ii}\left(t\right)$ refers to the inertial mass of i-th particles, and $rand_{i}$ indicates an arbitrary integer $\in \left[0,\;1\right].$

- Step seven: Upgrade particle location

Here, the location of i-th particles in the d-th direction, $p_{i}^{d}\left(t+1\right)$, is evaluated by the following expression:

(30) $$p_{i}^{d}\left(t+1\right)=p_{i}^{d}\left(t\right)+u_{i}^{d}\left(t+1\right)$$

- Step eight: Repeat steps two to seven until the end condition is met

In the EGSO algorithm, the GSO algorithm is integrated into the cat map concept to reduce the ergodic problem, avoid premature convergence and enhance the algorithm’s efficiency. In optimization algorithms, the grouping of SI optimization algorithms and chaotic mapping improves the outcomes of SI optimization. This concept was confirmed by several instances and depends on Chaos features including sensitivity, randomness, and ergodicity to the initial condition. Hence, an attempt was made to integrate the Chaos concept with GSO to attain good accuracy and efficiency [23]. Until now, the SI optimization approach was generally integrated into tent or logistic mapping models to accomplish a certain amount of progress. However, the chaotic sequence produced from the logistic mapping is of a non-uniform distribution. This follows Chebyshev’s Distribution, where density is lower at the center and is higher at both ends. These features have some level of impact on the ergodicity of optimization solution space. The chaotic sequence made from tent mapping models follows uniform distribution; however, the values rapidly fall into the smaller cycle due to the impact of word length. It therefore lacks better randomness. This method could generate a uniformly distributed chaotic sequence, to enhance GSO single distribution and might improve the model searching efficacy. A chaotic algorithm, Cat Map, is generally utilized in image encryption.

(31) $${\begin{array}{c} x_{n+1}=\left(x_{n}+y_{n}\right)\\ y_{n+1}=\left(x_{n}+2y_{n}\right) \end{array}}_{mod1}$$

Here, the chaotic sequence produced by the higher dimensional Cat Map at the range of [0, 1] is fixed as an arbitrary value rather than the rand function to determine individual distribution. The mapping value lies within the range of [0, 1] when building the multi-dimension chaotic sequence Cat(i,j). The sequential dataset in the dimension is independent of others and has stronger ergodicity. These features decrease the ergodic problem carried by random distribution and assures the ergodicity of individuals flying in the solution space. This technique might improve the algorithm performance and prevent premature convergence.

## 3. Results and Discussion

This section describes the experimental validation of the EGSO-HDLM technique, which was carried out with the help of the benchmark epidemiology dataset from Kaggle repository [24]. In this article, a set of 5000 samples in positive class and 5000 samples in negative classes were considered. The proposed model was simulated using the Python 3.6.5 tool on PC i5-8600k, GeForce 1050Ti 4 GB, 16 GB RAM, 250 GB SSD, and 1 TB HDD. The parameter settings were as follows: dropout: 0.5, batch size: 5, epoch count: 50, and activation: ReLU.

**Figure 3:**
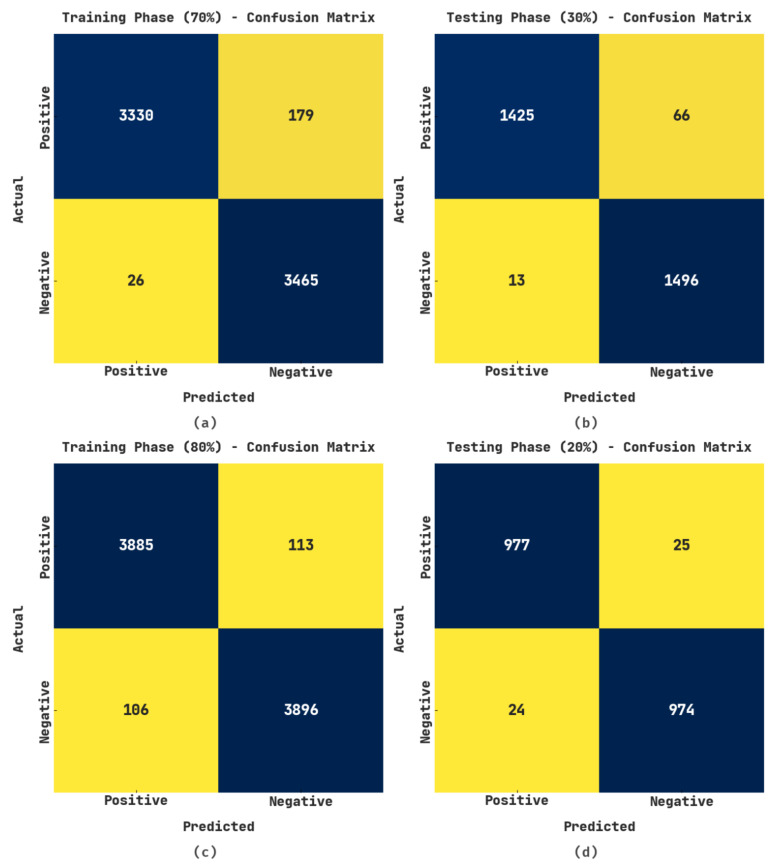
Confusion matrices of EGSO-HDLM algorithm (**a**) 70% of TR; (**b**) 30% of TS; (**c**) 80% of TR; (**d**) 20% of TS data.

The confusion matrices generated by the EGSO-HDLM model on the test data are demonstrated in Figure 3. For 70% of the training (TR) data, the EGSO-HDLM model identified 3330 samples in the positive class and 3465 samples in the negative class. Additionally, for 30% of the testing (TS) data, the EGSO-HDLM system identified 1425 samples in the positive class and 1496 samples in the negative class. In addition, for 80% of the TR data, the EGSO-HDLM method identified 3885 samples as positive class and 3896 samples as negative class. Finally, on 20% of TS data, the EGSO-HDLM system identified 977 samples in the positive class and 974 samples in the negative class.

**Figure 4:**
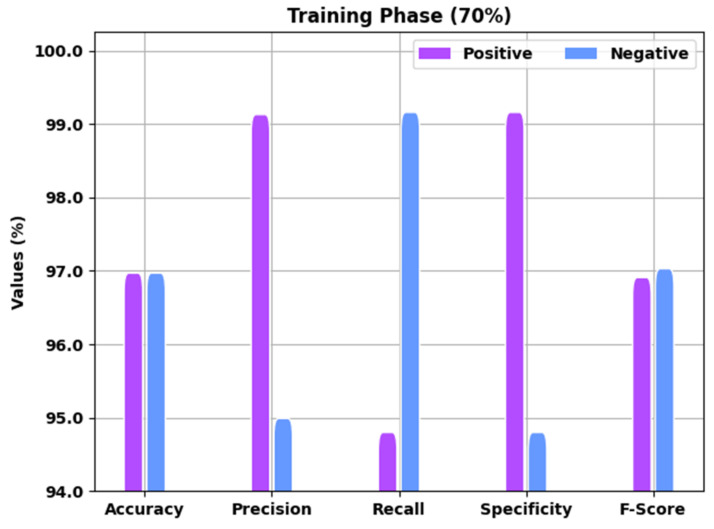
Result analysis of EGSO-HDLM approach under 70% of TR data.

Table 1 offers detailed COVID-19 recognition outcomes of the EGSO-HDLM model for 70% of the TR and 30% of the TS data. A brief result analysis of the EGSO-HDLM model in the identification of COVID-19 for 70% of the TR data is depicted in Figure 4. The results implied that the EGSO-HDLM model classified all the samples into corresponding positive and negative classes. For instance, the EGSO-HDLM model identified positive samples with $accu_{y}$ of 97.07%, $prec_{n}$ of 99.23%, $reca_{l}$ of 94.90%, $spec_{y}$ of 99.26%, and $F_{score}$ of 97.01%. Additionally, the EGSO-HDLM technique identified negative samples with $accu_{y}$ of 97.07%, $prec_{n}$ of 95.09%, $reca_{l}$ of 99.26%, $spec_{y}$ of 94.90%, and $F_{score}$ of 97.13%.

**Figure 5:**
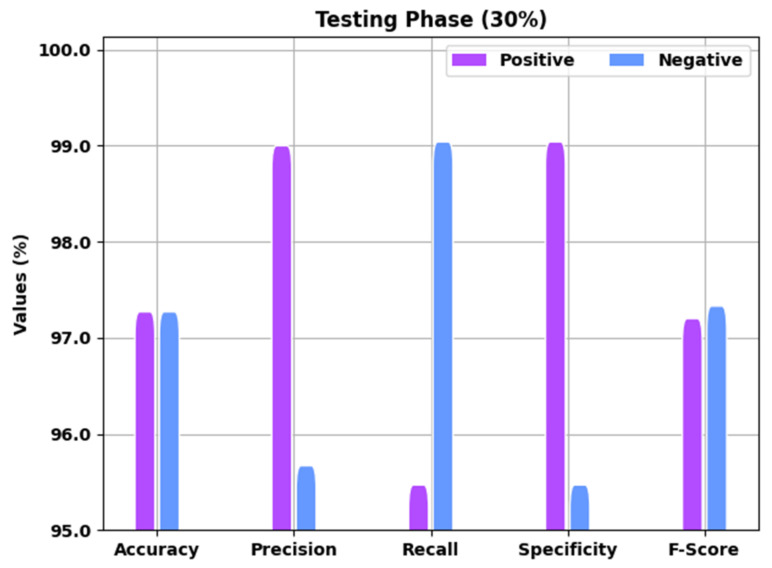
Result analysis of EGSO-HDLM approach under 30% of TS data.

A brief overview of the EGSO-HDLM technique in the identification of COVID-19 under 30% the TS data is shown in Figure 5. The results implied that the EGSO-HDLM methodology recognized all the samples as corresponding positive and negative classes. For example, the EGSO-HDLM system identified positive samples with $accu_{y}$ of 97.37%, $prec_{n}$ of 99.10%, $reca_{l}$ of 95.57%, $spec_{y}$ of 99.14%, and $F_{score}$ of 97.30%. Moreover, the EGSO-HDLM approach identified negative samples with $accu_{y}$ of 97.37%, $prec_{n}$ of 95.77%, $reca_{l}$ of 99.14%, $spec_{y}$ of 95.57%, and $F_{score}$ of 97.43%.

**Figure 6:**
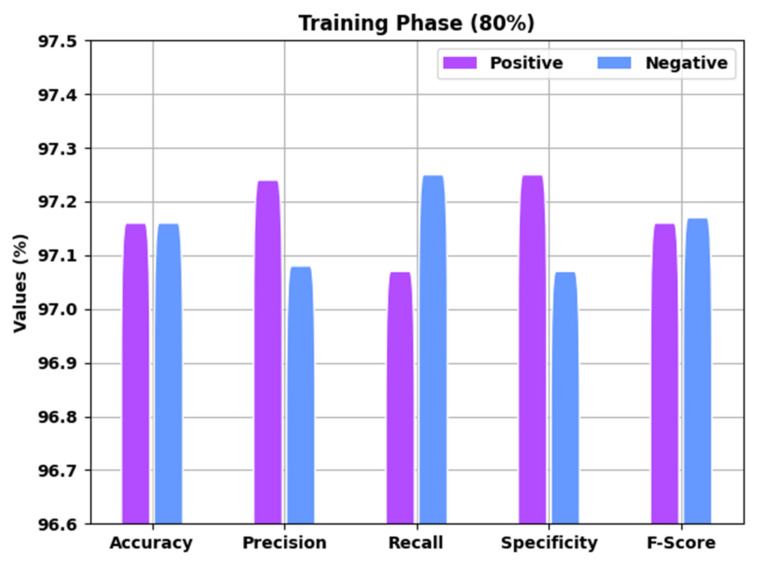
Result analysis of EGSO-HDLM approach under 80% of TR data.

Table 2 presents detailed COVID-19 identification outcomes of the EGSO-HDLM system for 80% of the TR and 20% of the TS data. A brief analysis of the EGSO-HDLM approach in the identification of COVID-19 for 80% of the TR data is portrayed in Figure 6. The results implied that the EGSO-HDLM model identified all the samples into corresponding positive and negative classes. For example, the EGSO-HDLM methodology identified positive samples with $accu_{y}$ of 97.26%, $prec_{n}$ of 97.34%, $reca_{l}$ of 97.17%, $spec_{y}$ of 97.35%, and $F_{score}$ of 97.26%. Additionally, the EGSO-HDLM model identified negative samples with $accu_{y}$ of 97.26%, $prec_{n}$ of 97.18%, $reca_{l}$ of 97.35%, $spec_{y}$ of 97.17%, and $F_{score}$ of 97.27%.

**Figure 7:**
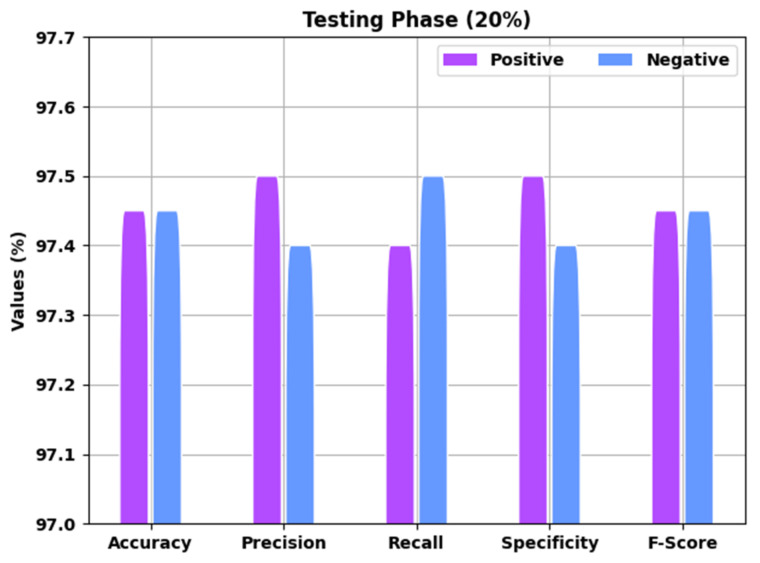
Result analysis of EGSO-HDLM approach under 20% of TS data.

A brief overview of the EGSO-HDLM approach in the identification of COVID-19 for 20% of the TS data is depicted in Figure 7. The results implied that the EGSO-HDLM technique identified all the samples as corresponding positive and negative classes. For example, the EGSO-HDLM method identified positive samples with $accu_{y}$ of 97.55%, $prec_{n}$ of 97.60%, $reca_{l}$ of 97.50%, $spec_{y}$ of 97.60%, and $F_{score}$ of 97.55%. Additionally, the EGSO-HDLM system identified negative samples with $accu_{y}$ of 97.55%, $prec_{n}$ of 97.50%, $reca_{l}$ of 97.60%, $spec_{y}$ of 97.50%, and $F_{score}$ of 97.55%.

**Figure 8:**
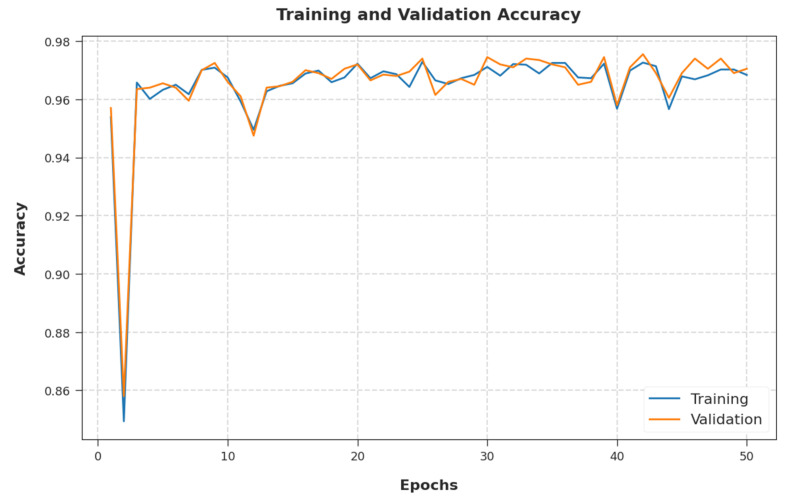
TA and VA analysis of EGSO-HDLM methodology.

The training accuracy (TA) and validation accuracy (VA) attained by the EGSO-HDLM method on test dataset are illustrated in Figure 8. The experimental outcome implied that the EGSO-HDLM system gained maximum values of TA and VA. Particularly, the VA seemed to be higher than TA.

**Figure 9:**
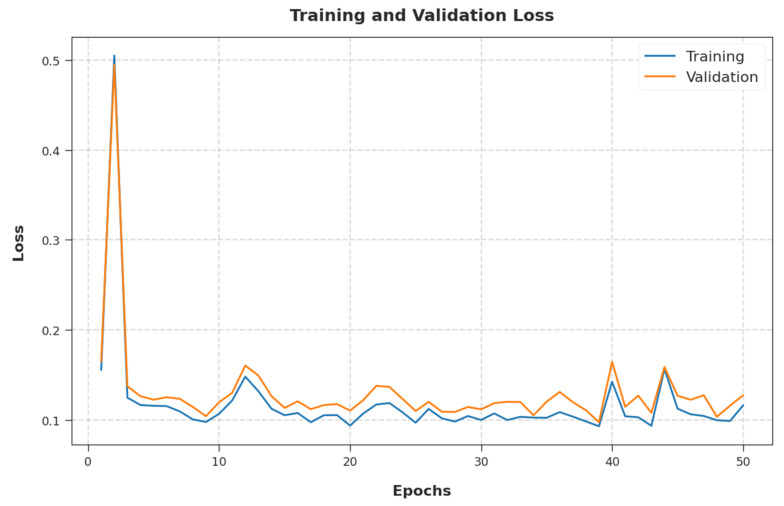
TL and VL analysis of EGSO-HDLM methodology.

The training loss (TL) and validation loss (VL) achieved by the EGSO-HDLM system on test dataset are established in Figure 9. The experimental outcome implied that the EGSO-HDLM methodology had the lowest values of TL and VL. In particular, the VL was lower than TL.

**Figure 10:**
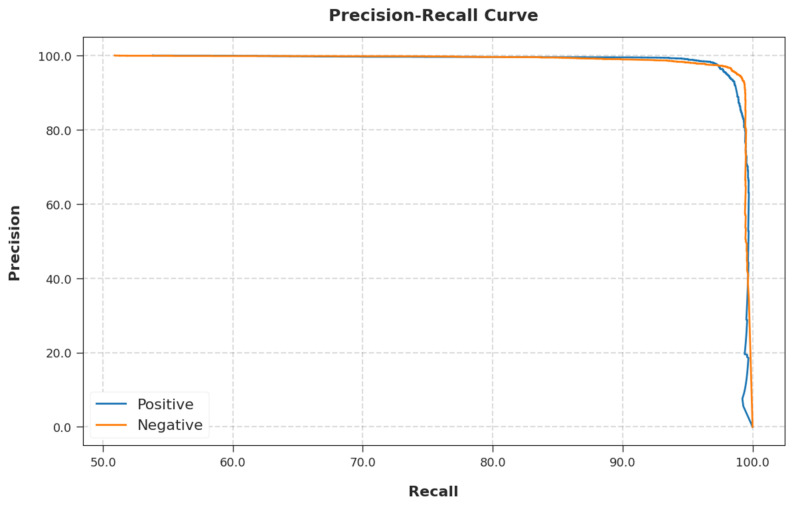
Precision-recall curve analysis of EGSO-HDLM methodology.

A brief precision-recall analysis of the EGSO-HDLM methodology on test dataset is portrayed in Figure 10. By observing the figure, it can be seen that the EGSO-HDLM technique accomplished maximum precision-recall performance under all classes.

**Figure 11:**
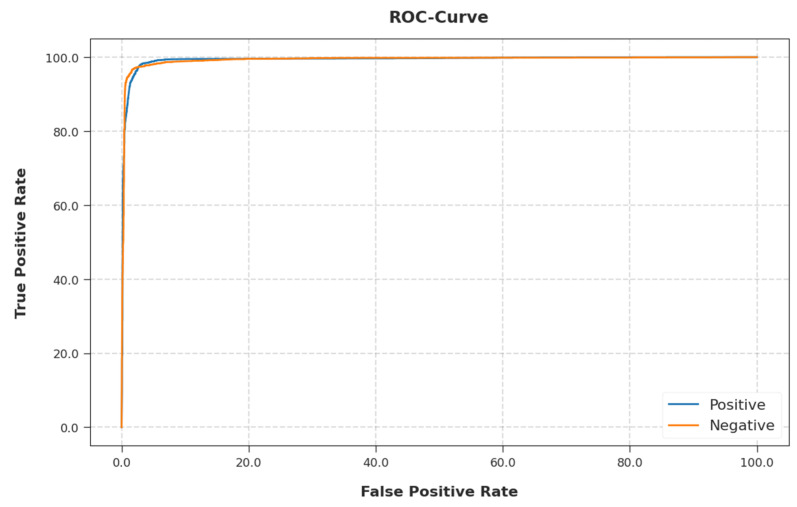
ROC curve analysis of EGSO-HDLM methodology.

A detailed ROC inquiry of the EGSO-HDLM algorithm on the test dataset is portrayed in Figure 11. The results implied that the EGSO-HDLM technique exhibited its ability in categorizing two distinct classes on the test dataset.

**Figure 12:**
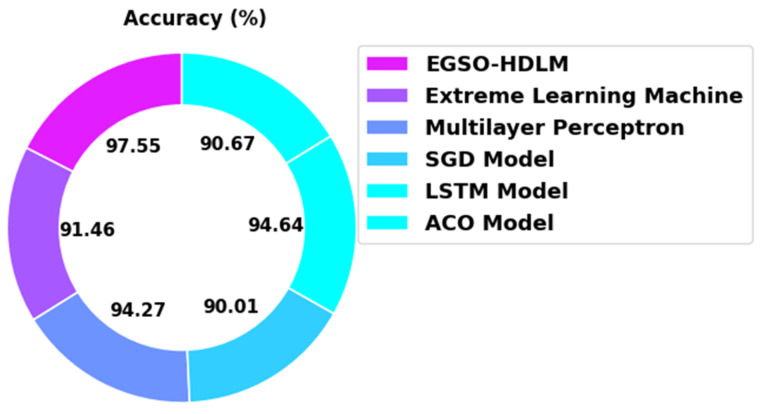
$Accu_{y}$ analysis of EGSO-HDLM algorithm with existing methodologies.

Table 3 portrays a detailed comparison study of the EGSO-HDLM model with recent models under several measures [16]. Figure 12 illustrates an extensive $accu_{y}$ in the comparison of the EGSO-HDLM model with existing models. The figure indicates that the SGD and ACO models displayed poor performance with minimal $accu_{y}$ values of 90.01% and 90.67%, respectively. Additionally, the ELM model obtained a slightly enhanced $accu_{y}$ value of 91.46%. Next, the MLP and LSTM models demonstrated a reasonably closer $accu_{y}$ of 94.27% and 94.64%, respectively. However, the EGSO-HDLM model offered a maximum $accu_{y}$ of 97.55%.

**Figure 13:**
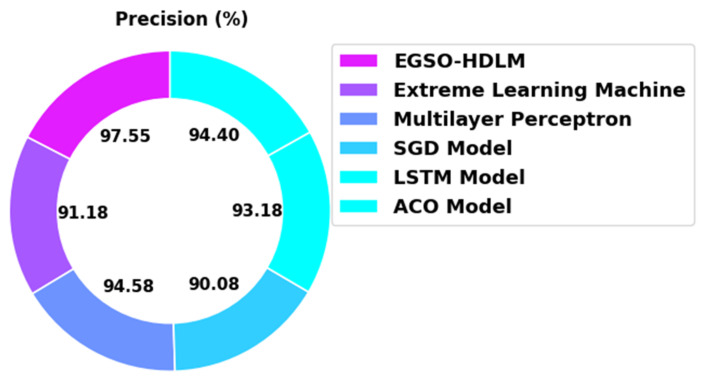
$Prec_{n}$ analysis of EGSO-HDLM algorithm with existing methodologies.

Figure 13 demonstrates an extensive $prec_{n}$ analysis of the EGSO-HDLM technique with existing models. The figure indicates that the SGD and ACO algorithms demonstrated poor performance with minimal $prec_{n}$ values of 90.08% and 94.40%, respectively. The ELM approach obtained a slightly enhanced $accu_{y}$ value of 91.18%. Next, the MLP and LSTM models demonstrated a reasonably closer $prec_{n}$ of 94.58% and 93.18% correspondingly. However, the EGSO-HDLM method provided the maximum $prec_{n}$ of 97.55%.

**Figure 14:**
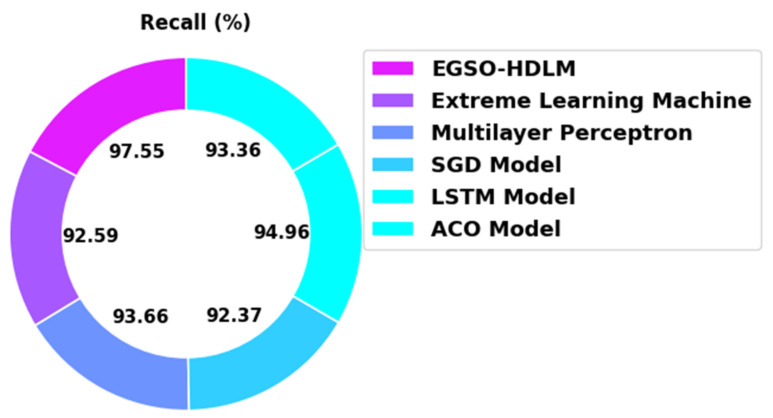
$Reca_{l}$ analysis of EGSO-HDLM algorithm with existing methodologies.

Figure 14 illustrates an extensive $reca_{l}$ inspection of the EGSO-HDLM algorithm in comparison with existing models. The figure indicates that the SGD and ACO systems demonstrated poor performance with minimal $reca_{l}$ values of 92.37% and 93.36%, respectively. Additionally, the ELM approach gained a slightly enhanced $accu_{y}$ value of 92.59%. Next, the MLP and LSTM models displayed a reasonably closer $reca_{l}$ of 93.66% and 94.96%, respectively. However, the EGSO-HDLM system offered the maximum $reca_{l}$ of 97.55%.

Therefore, the experimental results indicated that the EGSO-HDLM model has the ability to classify COVID-19 in an epidemiology dataset. The enhanced performance of the proposed model is due to the fusion process and EGSO-based hyperparameter tuning process.

## 4. Conclusions

In this study, a new EGSO-HDLM model was designed for the identification and classification of COVID-19 using epidemiology data. Primarily, the presented EGSO-HDLM model applied data normalization to pre-process the actual data. Then, the HCNN-GRUF model was applied for the classification process. Moreover, the hyperparameter optimization of the HCNN-GRUF model was improved by the use of the EGSO algorithm, which was derived by including the concepts of cat map and the traditional GSO algorithm. The design of the EGSO algorithm helps in reducing the ergodic problem, avoiding premature convergence, and enhancing algorithm efficiency. To assure the better performance of the EGSO-HDLM model, an extensive set of experimental validations on a benchmark dataset was performed. The simulation results ensured the enhanced performance of the EGSO-HDLM model over recent approaches. In future, the classification performance of the EGSO-HDLM model can be improved by the use of feature selection and outlier removal approaches. Moreover, the proposed model can be extended to the detection of other diseases.

## Figures and Tables

**Table 1:** Result analysis of EGSO-HDLM technique with various measures for 70% of TR and 30% of TS data.

Class Labels	Accuracy	Precision	Recall	Specificity	F-Score
Training Phase (70%)					
Positive	97.07	99.23	94.90	99.26	97.01
Negative	97.07	95.09	99.26	94.90	97.13
Average	97.07	97.16	97.08	97.08	97.07
Testing Phase (30%)					
Positive	97.37	99.10	95.57	99.14	97.30
Negative	97.37	95.77	99.14	95.57	97.43
Average	97.37	97.44	97.36	97.36	97.37

**Table 2:** Result analysis of EGSO-HDLM technique with various measures for 80% of TR and 20% of TS data.

Class Labels	Accuracy	Precision	Recall	Specificity	F-Score
Training Phase (80%)					
Positive	97.26	97.34	97.17	97.35	97.26
Negative	97.26	97.18	97.35	97.17	97.27
Average	97.26	97.26	97.26	97.26	97.26
Testing Phase (20%)					
Positive	97.55	97.60	97.50	97.60	97.55
Negative	97.55	97.50	97.60	97.50	97.55
Average	97.55	97.55	97.55	97.55	97.55

**Table 3:** Comparative analysis of EGSO-HDLM technique with existing approaches [14].

Methods	Accuracy	Precision	Recall
EGSO-HDLM	97.55	97.55	97.55
Extreme Learning Machine	91.46	91.18	92.59
Multilayer Perceptron	94.27	94.58	93.66
SGD Model	90.01	90.08	92.37
LSTM Model	94.64	93.18	94.96
ACO Model	90.67	94.40	93.36

## Data Availability

Data sharing is not applicable to this article as no datasets were generated during the current study.
